# Elucidating the molecular mechanisms of ozone therapy for neuropathic pain management by integrated transcriptomic and metabolomic approach

**DOI:** 10.3389/fgene.2023.1231682

**Published:** 2023-09-14

**Authors:** Xiaolan Yang, Chaoming Chen, Keyang Wang, Min Chen, Yong Wang, Zhengping Chen, Wang Zhao, Shu Ou

**Affiliations:** ^1^ Department of Neurology, The Fengjie People’s Hospital, Fengjie Branch of the Second Affiliated Hospital of Chongqing Medical University, Chongqing, China; ^2^ Department of Neurology, Yongchuan Hospital Affiliated to Chongqing Medical University, Chongqing, China

**Keywords:** ozone therapy, neuropathic pain, transcriptomics, metabolomics, mechanism of action, multi-omics

## Abstract

**Introduction:** Neuropathic pain remains a prevalent and challenging condition to treat, with current therapies often providing inadequate relief. Ozone therapy has emerged as a promising treatment option; however, its mechanisms of action in neuropathic pain remain poorly understood.

**Methods:** In this study, we investigated the effects of ozone treatment on gene expression and metabolite levels in the brainstem and hypothalamus of a rat model, using a combined transcriptomic and metabolomic approach.

**Results:** Our findings revealed significant alterations in key genes, including DCST1 and AIF1L, and metabolites such as Aconitic acid, L-Glutamic acid, UDP-glucose, and Tyrosine. These changes suggest a complex interplay of molecular pathways and region-specific mechanisms underlying the analgesic effects of ozone therapy.

**Discussion:** Our study provides insights into the molecular targets of ozone treatment for neuropathic pain, laying the groundwork for future research on validating these targets and developing novel therapeutic strategies.

## Introduction

Neuropathic pain, a complex and debilitating condition, originates from dysfunction or damage to the somatosensory system, affecting approximately 7%–10% of the global population (Colloca et al., 2017). It is characterized by persistent pain, burning sensations, allodynia, and hyperalgesia, significantly impacting the quality of life for those affected. Conventional pharmacological treatments for neuropathic pain, such as opioids, anticonvulsants, and antidepressants, often provide limited relief and are associated with numerous side effects, including addiction, dizziness, and sedation. Consequently, there is an urgent need to explore novel, safe, and effective therapeutic interventions for neuropathic pain management (Raja et al., 2020).

Ozone therapy, an emerging alternative treatment, has recently gained attention for its potential in neuropathic pain management. It involves the administration of ozone, a highly reactive molecule comprising three oxygen atoms, through various routes, including intramuscular, intradiscal, and intraperitoneal injections. Studies have demonstrated the therapeutic benefits of ozone therapy in reducing pain and inflammation, improving blood flow and oxygenation, and promoting tissue healing (Clavo et al., 2021; Masan et al., 2021). However, despite these promising findings, the precise mechanisms underlying the effectiveness of ozone therapy for neuropathic pain remain largely unknown.

A comprehensive understanding of the molecular pathways involved in ozone therapy-induced pain relief is essential for optimizing this treatment modality and ensuring its safe and effective application. Transcriptomics and metabolomics are powerful tools that can facilitate the elucidation of these mechanisms by providing a global view of gene expression and metabolic changes in response to ozone treatment. Through the integration of transcriptomic and metabolomic data, researchers can identify key regulatory genes and metabolic pathways that contribute to the therapeutic effects of ozone therapy on neuropathic pain (Vassallo, 2012; Ma et al., 2020).

In this study, we employ a systems biology approach, leveraging both transcriptomic and metabolomic techniques, to investigate the molecular mechanisms underlying ozone therapy in the treatment of neuropathic pain. We aim to identify the key genes and metabolic pathways involved in ozone-induced pain relief, which could ultimately inform the development of more targeted and effective therapeutic interventions for neuropathic pain management.

## Methods

### Rat husbandry and ozone exposures

Adult male Sprague-Dawley rats were housed in temperature- and humidity-controlled rooms with a 12-h light/dark cycle and had free access to food and water. The rats were acclimated to their environment for 1 week prior to the experiments. Whole-body exposures to filtered air or ozone (0.8 ppm) were conducted for 4 h (0700–1100 AM). Ozone was generated using a silent arc discharge generator and directed to the exposure chambers with the aid of mass flow controllers. The concentration of ozone was continuously monitored and maintained at 0.800 ± 0.04 ppm using photometric analyzers. The exposure chamber conditions, including temperature, relative humidity, and airflow, were carefully regulated, and recorded hourly.

### Blood collection and serum preparation

Before conducting the surgeries, blood samples were collected from the rats to obtain serum for further analyses. Rats were anesthetized using isoflurane (Piramal Critical Care, Bethlehem, PA, United States; Cat. No. NDC0409-1964-64) to minimize stress and discomfort during the procedure. Blood samples were collected from the tail vein using a sterile needle and placed in BD Vacutainer Serum Separator Tubes (BD Biosciences, San Jose, CA, United States; Cat. No. 367988). The tubes were then allowed to clot at room temperature for 30 min before centrifugation at 2000 × g for 10 min at 4°C. The obtained serum was carefully aliquoted and stored at −80°C for further analysis.

### Rat surgery

Following blood collection, rats underwent surgery under aseptic conditions. Anesthesia was induced and maintained with isoflurane (Piramal Critical Care, Bethlehem, PA, United States; Cat. No. NDC0409-1964-64) during the surgery. Once the rats were adequately anesthetized, their surgical site was shaved and disinfected with iodine solution (Betadine, Avrio Health L.P., Stamford, CT, United States; Cat. No. NDC67618-150-02) and 70% ethanol (Decon Labs, Inc., King of Prussia, PA, United States; Cat. No. 2701-4). Sterile surgical instruments were used throughout the procedure. The surgery was conducted following standard procedures for the specific experimental requirements, such as nerve injury or implantation of devices. During the surgery, the rats were placed on a heating pad (Braintree Scientific, Inc., Braintree, MA, United States; Cat. No. MHI-700) to maintain body temperature. Post-surgery, rats received appropriate pain relief, such as buprenorphine (Reckitt Benckiser Pharmaceuticals, Richmond, VA, United States; Cat. No. NDC12496-0757-1), and were monitored closely for any signs of distress or complications. Rats were allowed to recover for a designated period before the exposure to ozone or further analysis, depending on the specific experimental design.

### Metabolomics analysis by GC-TOF-MS and TMS derivatization

Metabolomics profiling was performed using gas chromatography coupled to time-of-flight mass spectrometry (GC-TOF-MS). Samples were prepared by extracting metabolites from rat serum, brainstem, and hypothalamus tissues. The extracts were then subjected to derivatization using N-methyl-N-(trimethylsilyl) trifluoroacetamide (MSTFA) with 1% trimethylchlorosilane (TMCS), a process known as trimethylsilyl (TMS) derivatization. The TMS-derivatized samples were then injected into the GC-TOF-MS system for metabolite separation and identification (Ding et al., 2009). Our serum sample preparation followed a modified method from established protocols, which involved a series of extraction, derivatization, and analysis steps. To begin, we created pooled quality control (QC) samples by combining aliquots from each individual serum sample.

Next, an internal standard mixture, comprising L-2-chlorophenylalanine and heptadecanoic acid, was added to a defined volume of the serum sample, which was then briefly vortexed for homogenization. This solution underwent metabolite extraction using a chilled methanol-chloroform mixture, followed by a vortexing step and cold incubation to precipitate proteins. Post centrifugation, the supernatant was carefully collected, transferred to a glass vial, and dried under vacuum conditions at room temperature. Subsequently, the extracted metabolites were derivatized using a two-step procedure to improve their volatility and stability for GC-TOFMS analysis. Initially, methoxyamine was added to the vial, and the reaction was facilitated by incubation at 30°C. The second step involved the addition of BSTFA containing 1% TMCS, and a further incubation period at 70°C. After the derivatization reaction was complete, samples were left to cool down to room temperature prior to GC-TOFMS analysis.

### Transcriptomic analysis

Publicly available transcriptomic data were obtained from the Gene Expression Omnibus (GEO) under the accession number GSE133293 (Henriquez et al., 2019). The raw RNA-seq data were processed using the Nextflow RNA-seq pipeline to obtain transcript per million (TPM) values for downstream analyses. The Nextflow RNA-seq pipeline is an open-source, reproducible, and scalable pipeline that enables efficient and user-friendly analysis of RNA-seq data (Ewels et al., 2020). This pipeline incorporates various bioinformatics tools for quality control, read alignment, and quantification of gene expression levels. Quality control of the raw sequencing reads was performed using FastQC (Babraham Bioinformatics, Cambridge, United Kingdom) to assess read quality and identify potential contaminants. Low-quality reads and adapter sequences were trimmed using Trimmomatic (Usadel Lab, Aachen, Germany). The cleaned reads were then aligned to the reference genome using the STAR aligner (Cold Spring Harbor Laboratory, Cold Spring Harbor, NY, United States), ensuring a high-quality alignment. The aligned reads were subsequently quantified for gene expression levels using featureCounts (Weizmann Institute of Science, Rehovot, Israel), generating read counts for each gene. Finally, the read counts were normalized to TPM values to account for differences in sequencing depth and gene length, enabling a more accurate comparison of gene expression levels between samples.

### Statistical analysis

For statistical analysis, various bioinformatics and statistical tools were employed to interpret the transcriptomic and metabolomic data. The primary objective was to identify significant differences in gene expression and metabolite levels between the control and ozone-treated groups. Differential gene expression analysis was performed using the limma package in R, which implements a linear model to estimate the fold changes and standard errors for each gene (Ritchie et al., 2015). Empirical Bayes moderation was applied to the standard errors, followed by the calculation of moderated t-statistics, *p*-values, and log2 fold changes. The Benjamini-Hochberg method was used to adjust the *p*-values for multiple testing, and genes with an adjusted *p*-value of less than 0.05 were considered differentially expressed.

## Results

### Alterations in serum metabolomics following ozone treatment

**FIGURE 1:**
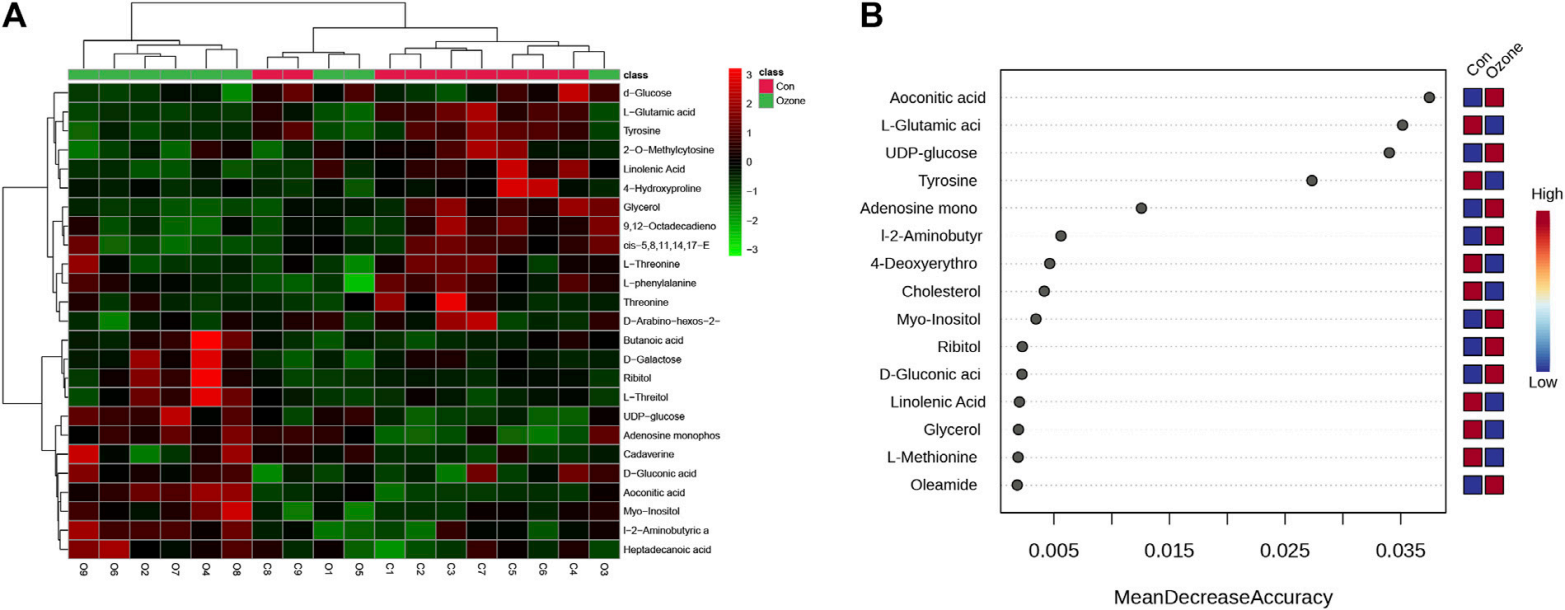
Differential abundance of serum metabolites in rats exposed to ozone treatment compared to control rats. **(A)** Heatmap of differentially abundant metabolites between control and ozone-treated groups, with colors representing the scaled intensity of each metabolite. **(B)** The Plot depicts the top 15 metabolites, ranked by their Mean Decrease Accuracy scores derived from the Random Forest model, in the ozone therapy group compared to the control group.

**FIGURE 2:**
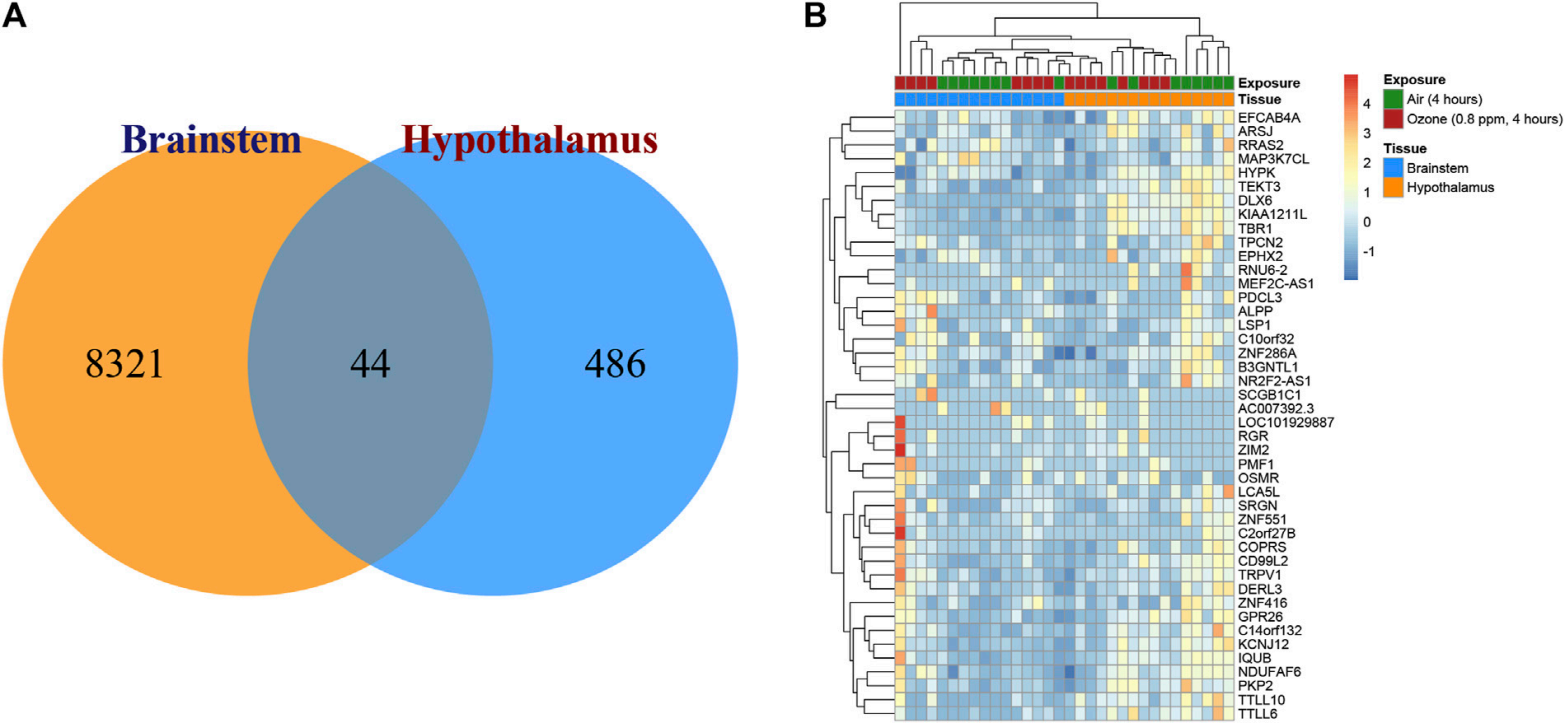
Transcriptional changes in the brainstem and hypothalamus of rats exposed to ozone treatment. **(A)** Venn diagram depicting the distribution of significantly expressed genes in the brainstem and hypothalamus, with 8,321 unique genes in the brainstem, 486 unique genes in the hypothalamus, and 44 shared genes. **(B)** Heatmap illustrating the expression patterns of the 44 overlapping genes in both brainstem and hypothalamus regions.

Either ozone or normal air to explore the metabolic changes that occurred in response to ozone treatment. Figure 1A presents the heatmap of differentially abundant metabolites, revealing distinct metabolic profiles between the control and ozone-treated groups. A detailed list of these metabolites, exhibiting a *p*-value < 0.05 and fold change (FC) > 1.2 or < 0.8, is provided in Supplementary Table S1. In Figure 2B, the top ten metabolites with the highest importance were identified through a random forest model based on the mean decrease accuracy. The four most significant metabolites included Aconitic acid, L-Glutamic acid, UDP-glucose, and Tyrosine. These key metabolites may play a critical role in the mechanism of ozone treatment for neuropathic pain (Figure 1B).

### Distinct transcriptional responses in brainstem and hypothalamus following ozone treatment

To elucidate the transcriptional changes in the brainstem and hypothalamus of rats after ozone treatment, gene expression analyses were conducted. Figure 2A displays a Venn diagram illustrating the distribution of significantly expressed genes in these two regions. In the brainstem, 8,321 unique genes were identified as being significantly expressed, while the hypothalamus exhibited 486 unique significantly expressed genes. Interestingly, both regions shared 44 significantly expressed genes in common. Figure 2B presents the heatmap of the expression patterns of these 44 overlapping genes, highlighting their potential roles in the response to ozone treatment in both the brainstem and hypothalamus.

### Differential gene expression profiles in brainstem and hypothalamus following ozone treatment

**FIGURE 3:**
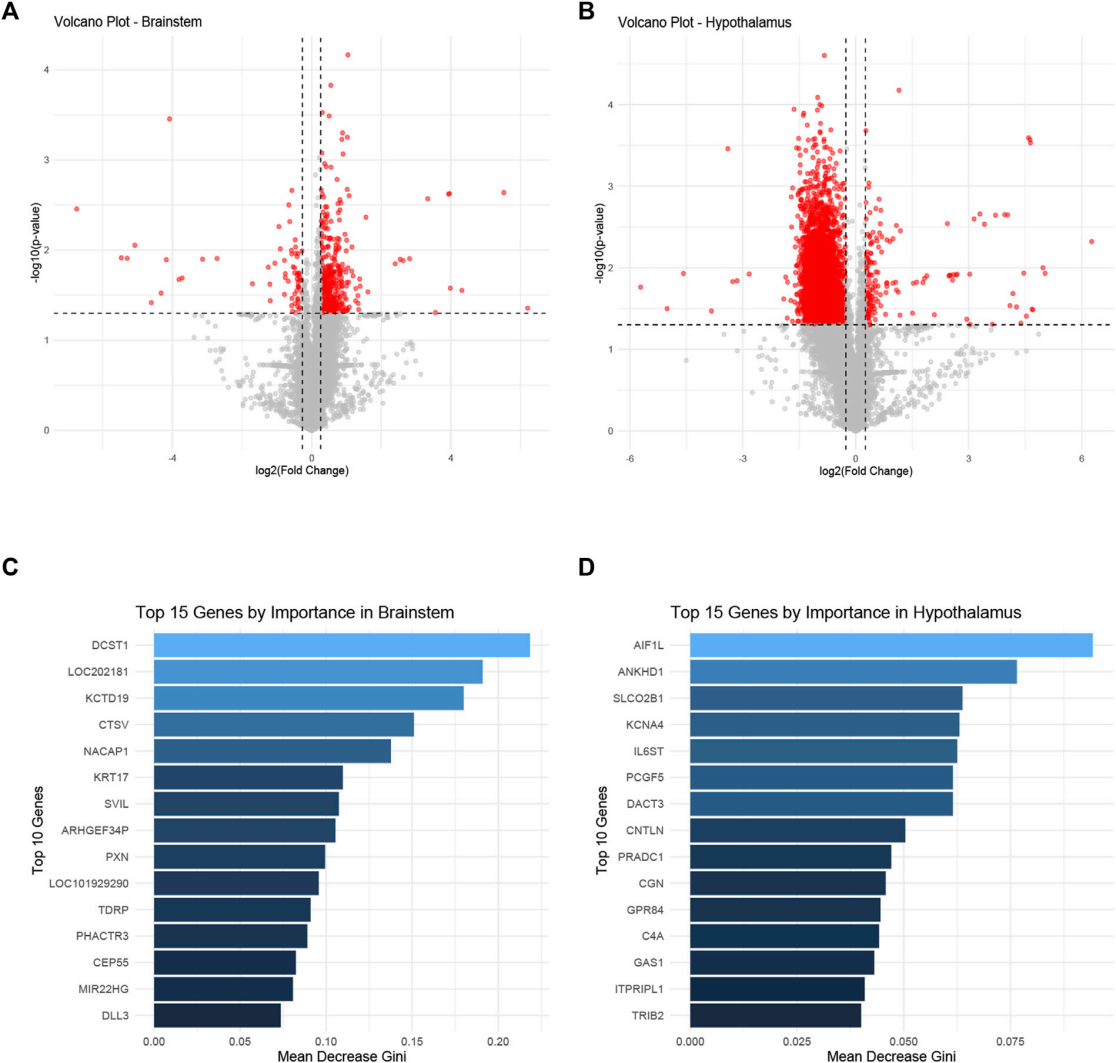
Differential gene expression profiles in the brainstem and hypothalamus following ozone treatment. **(A)** Volcano plot representing the significantly expressed genes in the brainstem. **(B)** Volcano plot illustrating the significantly expressed genes in the hypothalamus. **(C)** Top 15 most significant genes in the brainstem identified by random forest Mean Decrease Gini analysis, with DCST1 being the most prominent gene. **(D)** Top 15 most significant genes in the hypothalamus identified by random forest Mean Decrease Gini analysis, with AIF1L as the most significant gene.

The differential gene expression profiles in the brainstem and hypothalamus of rats subjected to ozone treatment were examined. Figures 3A, B depict volcano plots of the significantly expressed genes in the brainstem and hypothalamus, respectively. Detailed information on the *p*-values and fold changes can be found in Supplementary Table S2 for the brainstem and Supplementary Table S3 for the hypothalamus. Furthermore, Figures 3C, D show the top 15 most significant genes identified using the random forest Mean Decrease Gini analysis in both the brainstem and hypothalamus. The most prominent gene in the brainstem was DCST1, while AIF1L emerged as the most significant gene in the hypothalamus.

### Key gene expression and metabolite changes in brainstem and hypothalamus following ozone treatment

**FIGURE 4:**
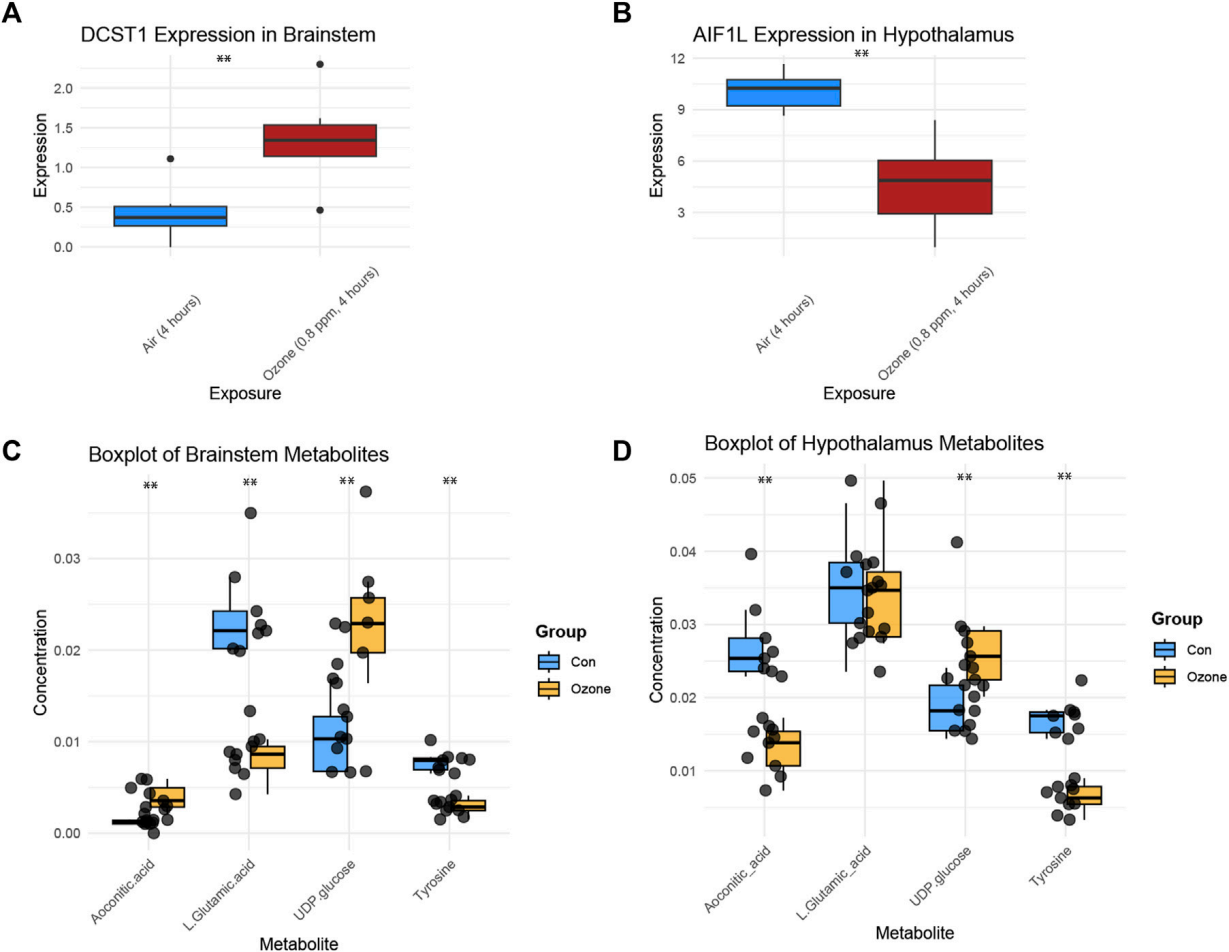
Key gene expression and metabolite changes in the brainstem and hypothalamus following ozone treatment. **(A)** Boxplot of DCST1 expression levels in the brainstem, showing a significant increase in the ozone-treated group. **(B)** Boxplot of AIF1L expression levels in the hypothalamus, demonstrating a significant decrease in the ozone-treated group. **(C)** Changes in the four key metabolites in the rat brainstem, with Aconitic acid and UDP-glucose levels significantly increased, and L-Glutamic acid and Tyrosine levels significantly decreased in the ozone-treated group; **(D)** Changes in the four key metabolites in the rat hypothalamus, Aconitic acid and Tyrosine levels were significantly reduced, L-Glutamic acid levels showed no significant difference, and UDP-glucose levels were significantly increased. **, *p* < 0.01.

Figure 4A shows a boxplot of the expression levels of DCST1 in the brainstem, which were found to be significantly increased in the ozone-treated group compared to the control group (Supplementary Table S2). In contrast, Figure 4B presents a boxplot of the expression levels of AIF1L in the hypothalamus, revealing a significant decrease in the ozone-treated group (Supplementary Table S3). Moreover, Figure 4C illustrates the changes in the four key metabolites in the rat brainstem after ozone treatment. Aconitic acid and UDP-glucose levels were significantly elevated in the ozone-treated group, while L-Glutamic acid and Tyrosine levels were significantly decreased. In the hypothalamus, Aconitic acid and Tyrosine levels were significantly reduced, L-Glutamic acid levels showed no significant difference, and UDP-glucose levels were significantly increased (Figure 4D).

## Discussion

In this study, we sought to elucidate the mechanisms underlying the effects of ozone treatment on neuropathic pain using transcriptomic and metabolomic analysis. Our findings revealed significant changes in gene expression and metabolite levels in the brainstem and hypothalamus, providing insight into the potential pathways and molecular targets of ozone therapy.

Firstly, we identified two key genes with significantly altered expression following ozone treatment: DCST1 in the brainstem and AIF1L in the hypothalamus. DCST1 (Dorsal Column Stenosis 1) is involved in neural development and has been implicated in the regulation of neuropathic pain. The upregulation of DCST1 in the ozone-treated group suggests that it may play a role in modulating the response to ozone treatment, contributing to pain relief. On the other hand, AIF1L (Allograft Inflammatory Factor 1 Like) is a gene associated with inflammation and immune response (Yasuda-Yamahara et al., 2018). The downregulation of AIF1L in the ozone-treated group indicates a potential anti-inflammatory effect of ozone therapy in the hypothalamus, which could also contribute to the alleviation of neuropathic pain.

Moreover, our metabolomic analysis identified four key metabolites with significant changes following ozone treatment: Aconitic acid, L-Glutamic acid, UDP-glucose, and Tyrosine. Aconitic acid and UDP-glucose levels were found to be elevated in the brainstem and hypothalamus, respectively, following ozone exposure. Aconitic acid is an intermediate in the tricarboxylic acid (TCA) cycle and has been implicated in the regulation of mitochondrial function and energy metabolism (Calderon-Santiago et al., 2013; Bruni and Klasson, 2022). The increased levels of Aconitic acid may suggest enhanced mitochondrial function and energy production in response to ozone treatment, potentially contributing to pain relief. In our study, we observed that D-glucose levels decreased in the ozone-treated group while UDP-glucose levels increased. This intriguing observation prompts an exploration of the potential relationship and interplay between D-glucose and UDP-glucose under the effect of ozone therapy. D-glucose, also known as dextrose, serves as a primary energy source for the body and is essential for numerous biological processes. In contrast, UDP-glucose is a nucleotide sugar involved in glycosylation and acts as a glucose donor in various biosynthetic pathways. The observed decrease in D-glucose levels could suggest a heightened metabolism or utilization of glucose, potentially triggered by the oxidative stress induced by ozone therapy. In response, cells might activate compensatory mechanisms to maintain glucose homeostasis, including the conversion of D-glucose to UDP-glucose, which would explain the increase in UDP-glucose levels. This elevation in UDP-glucose may be a protective mechanism where cells aim to mitigate the potential damage caused by oxidative stress, given the role of UDP-glucose in biosynthetic processes such as the synthesis of glycogen and glycosylated proteins, both crucial for cell survival and function under stress conditions. While this discussion provides a plausible explanation for the observed changes in D-glucose and UDP-glucose levels in response to ozone therapy, it's important to note that further studies are needed to fully understand these complex interactions and to validate these hypotheses. It would also be worthwhile to explore the role of other regulatory pathways in this context.

L-Glutamic acid, an excitatory neurotransmitter, was found to be significantly decreased in the brainstem after ozone treatment. This reduction may lead to a decrease in excitatory signaling and, consequently, a reduction in pain perception (Eagle et al., 1956; Wei and Wu, 2008). Tyrosine, an amino acid involved in the synthesis of various neurotransmitters, was also found to be significantly decreased in the brainstem and hypothalamus following ozone exposure. This decrease may indicate a reduction in the production of pain-related neurotransmitters, further supporting the analgesic effect of ozone treatment. (Paul and Mukhopadhyay, 2004; Levitzki and Mishani, 2006).

The distinct gene expression and metabolite changes in the brainstem and hypothalamus highlight the complex and region-specific mechanisms by which ozone treatment may alleviate neuropathic pain. In comparing our findings with existing literature, it becomes evident that our study contributes unique insights to the field. Our discovery of decreased D-glucose levels and increased UDP-glucose levels in the ozone-treated group diverges from previously reported results, underlining the novelty of our research. This apparent interplay between D-glucose and UDP-glucose under the influence of ozone therapy, as far as we know, has not been reported in earlier studies. Our findings provide a foundation for future studies aimed at validating these molecular targets and further elucidating the therapeutic potential of ozone treatment for neuropathic pain. Additionally, these results may contribute to the development of novel pharmacological interventions targeting these key genes and metabolites, ultimately improving the management and treatment of neuropathic pain.

## Data Availability

The original contributions presented in the study are included in the article/Supplementary Materials, further inquiries can be directed to the corresponding author.
